# Exosomes loaded with programmed death ligand-1 promote tumor growth by immunosuppression in osteosarcoma

**DOI:** 10.1080/21655979.2021.1996509

**Published:** 2021-12-04

**Authors:** Lei Zhang, Lili Xue, Yanjuan Wu, Qilong Wu, Hongwei Ren, Xiang Song

**Affiliations:** a Oncology Department, The Second Hospital of Shanxi Medical University, China; b Cardio-Thoracic Surgery, The Second Hospital of Shanxi Medical University, China

**Keywords:** Exosomes, PD-l1, osteosarcoma, T cells

## Abstract

Osteosarcoma (OS) is a malignant tumor commonly observed in adolescents, who experience relapse and metastasis (30% of the total cases). Its progression is attributed to immune escape mediated by immune checkpoints. However, the intercellular connection between tumor cells and T cells remain unclear. This study was conducted to explore the effects of PD-L1-loaded exosomes on the tumor growth of OS. The exosomes were extracted from cells and tissues through ultracentrifugation. IFN-γ production was determined to evaluate the activity of Jurkat cells. The in vivo growth of OS cells was examined using a C3H xenograft model in mice, tumor volumes were monitored, and the proportion of CD3 + T cells in tumor tissues was detected. Results revealed that PD-L1 was significantly upregulated in the OS cell lines. MG63 and Saos-2 cells were the most abundant in PD-L1, so they were selected as investigation targets. PD-L1 was found to be also highly expressed in the exosomes isolated from MG63 and Saos-2 cells. The exosomes elicited significant inhibitory effects on IFN-γ secretion in Jurkat cells, which were abolished by the PD-L1 antibody or siRNAs. The in vivo growth of C3H cells was significantly facilitated by the overexpression of mPD-L1 or by the administration of mPD-L1-overloaded exosomes. The infiltration of CD3 + T cells was also decreased. The exosomes extracted from clinical PD-L1-positive OS tissues showed a promising inhibitory property against activated T cells. Therefore, PD-L1-loaded exosomes extracted from OS cells aggravated OS progression by suppressing T cell activities.

## Graphical abstract

**Figure:**
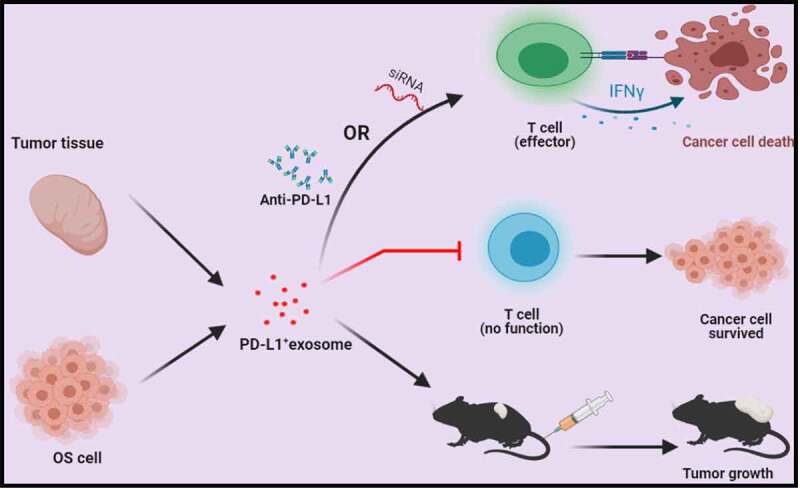

## Introduction

Osteosarcoma (OS) is a malignant tumor commonly observed in adolescents and accounts for approximately 36% of the population of patients with primary bone cancer [1]. Although OS therapies have been greatly improved and the survival rate of patients with OS has been significantly prolonged, relapse or metastasis is still observed in over 30% of patients [2,3]. Therefore, novel therapies for alleviating the prognosis of these patients should be developed. Recently, targeted therapy has been widely applied in the field of malignant tumors by directly inhibiting oncogenes or proteins to suppress cancer progression. In a tumor microenvironment, tumor-infiltrating lymphocytes are important components of tumor immunity. T cells are the main tumor effector cells activated or inactivated by costimulatory signals from antigen-presenting cells or tumor cells [4,5]. Dysfunction in T cells can be induced after they receive inhibitory signals; consequently, malignant tumors evade the immune system [6,7]. Programmed death ligand 1 (PD-L1) is the third B7 family member closely associated with T cell activation [8]. T cell immunoreaction mediated by PD-L1 is bidirectional. When CD28 is not involved in costimulation, PD-L1 provides a second positive signal of costimulation. However, when excessive T cell proliferation is induced by the signals obtained from CD3 and CD28, T cell activity is inhibited by the binding between PD-L1 and programmed death-1 (PD-1) [9]. In an immunohistochemical research on OS tissues from 69 patients, the positive rate of PD-L1 is 43.5%, and PD-L1 expression is negatively related to the survival of patients with OS [10]. In a PD-L1-knocked out OS cell line, malignancy is attenuated, and sensitivity against chemotherapy is increased [11]. Therefore, the underlying immune evasion mechanism of PD-L1 and the corresponding OS therapies should be explored.

Exosomes are a group of extracellular vesicles released by cells at a diameter of 30–150 nm; they are commonly observed in the supernatant of endothelial cells, lymphocytes, mesenchymal cells, dendritic cells, fibroblasts, and tumor cells [12]. They maintain communication among different cell types by carrying abundant substances from maternal cells, such as nucleic acids, proteins, and saccharides [13]. For instance, PD-L1 carried by exosomes from tumor cells plays a critical role in the immune escape of malignant tumors and contributes to the proliferation and metastasis of these tumors [14]. Immune checkpoint ligands, such as PD-L1 and FasL, are observed in the membrane of exosomes from tumor cells, and they induce apoptosis and dysfunction by binding to receptors on the membrane of targeted immune cells [15]. The immune evasion mechanism mediated by exosomes carrying PD-L1 has been widely reported in multiple types of malignant tumors, such as nonsmall cell lung cancer [16], breast carcinoma [17], and gastric carcinoma [18], but this mechanism is rarely reported in OS. In the present study, the immune evasion mechanism mediated by exosomes carrying PD-L1 in OS was investigated to explore the potential therapeutic target for OS and the effects of PD-L1-loaded exosomes on the tumor growth of OS.

## Materials and methods

### Cell lines and treatments

Human osteoblasts (hFOB 1.19 cells), OS cell lines (MG63, HOS/MNNG, Saos-2, SJSA-1, U2OS, SW1353, 143B, T1-73, and KHOS/NP), mouse OS cell line (C3H cells), and Jurkat cells were obtained from ATCC (Manassas, VA, USA) and cultured in DMEM supplemented with 10% FBS, 100 U/mL penicillin (Sigma, Missouri, USA), and 0.1 mg/mL streptomycin (Sigma, Missouri, USA) under the conditions of 5% CO_2_ and 37°C. The inflammatory state of Jurkat cells was induced by adding 50 ng/mL PMA (Sigma, Missouri, USA) and 500 ng/mL ionomycin (Sigma, Missouri, USA) for 6 h.

### Western blot assay

Total proteins were extracted from the cells or exosomes by using a lysis buffer (Cell Signaling Technology, California, USA) and quantified with a BCA kit (Shanghai Ze Ye Biotechnology Co., Ltd., Shanghai, China). Approximately 30 μg of proteins was loaded, separated through 12% SDS PAGE, and transferred to a PVDF membrane (Cell Signaling Technology, California, USA). Then, the protein-loaded PVDF membrane was mixed with 5% skim milk to block nonspecific binding proteins and incubated with a solution of primary antibodies against PD-L1 (1:800, R&D, Minnesota, USA), PD-L2 (1:800, R&D, Minnesota, USA), GRP 94 (1:800, R&D, Minnesota, USA), HSP70 (1:800, R&D, Minnesota, USA), HSP90 (1:800, R&D, Minnesota, USA), CD9 (1:800, R&D, Minnesota, USA), CD81 (1:800, R&D, Minnesota, USA), PD-1 (1:800, R&D, Minnesota, USA), and GADPH (1:800, R&D, Minnesota, USA) [19]. The membrane was subsequently incubated with the secondary antibody (R&D, Minnesota, USA). Lastly, bands were visualized with ECL kits (Cell Signaling Technology, California, USA), and the relative expression levels of target proteins were quantified with Image J software.

### Exosome extraction

After the cells (MG63, Saos-2, and hFOB 1.19 cells) were incubated for 48 h, the medium was changed with serum-free DMEM, incubated for another 48 h, and successively centrifuged at 300 × *g* for 10 min, 2000 × *g* for 10 min, and 10,000 × *g* for 30 min at 4°C. Subsequently, the supernatants were collected, centrifuged at 100,000 × *g* for 70 min at 4°C, washed, and resuspended with PBS buffer. The extracted exosomes were stored at −80°C for the subsequent experiments.

### Nanoparticle tracking analysis

The size distribution and concentration of the extracted exosomes were determined with a NanoSight NS300 instrument (Malvern, UK) and analyzed using the nanoparticle tracking analysis software (Malvern, UK).

### Transmission electron microscopy for the identification of the extracted exosomes

The extracted exosomes were fixed with 2.5% glutaraldehyde at 4°C for 12 h, loaded onto formvar/carbon-coated grids, and stained with aqueous phosphotungstic acid for several minutes. Their images were taken using a transmission electron microscope (Laird, Missouri, USA).

### ELISA assay

IFN-γ secretion by Jurkat cells was determined using commercially available kits (Elabscience, Wuhan, China). Briefly, standards were diluted to five gradient concentrations and added to the supernatants collected from each cultural medium in 96-well plates. After incubation for 30 min at 37°C, the samples were washed using a washing solution. Subsequently, the wells were added with conjugate reagents and incubated at 37°C for 30 min. Afterward, a TMB solution was added for coloration at 37°C for 15 min, and the stop solution was added to terminate the reaction. Lastly, the absorbance at 450 nm was detected with a microplate reader (Mindray, Shenzhen, China). IFN-γ/Control (%) was calculated using the following formula: IFN-γ/Control (%) = IFN-γ _test_/IFN-γ _PMA + ionomycin_.

### Knockdown of hPD-L1 in MG63 cells and overexpression of mPD-L1 in C3H cells

Two siRNAs targeting the human PD-L1 were specially designed to knock down the expression level of PD-L1 in MG63 cells. The following sequences were used: 5'-CACCGTCTTTATATTCATGACCTAC-3' for siRNA #1 and 5'-AAACGTAGGTCATGAATATAAAGAC-3' for siRNA #2. The siRNAs were carried by a lentiviral vector (lentiCRISPR v2, Addgene, Massachusetts, USA). Together with Lipofectamine 3000 (Invitrogen, Massachusetts, USA), they were transfected into MG63 cells for 48 h to obtain PD-L1-knocked down MG63 cells. A pGIPZ-shmPD-L1/Flag-mPD-L1 (mPD-L1) dual expression construct was transfected into C3H cells to downregulate the endogenous mPD-L1 expression, reconstitute Flag-mPD-L1 expression, and establish mPD-L1-overexpressing C3H cells in accordance with previously described methods [20].

### CCK-8 assay

In brief, C3H cells were added with 10 µL of CCK-8 solution and incubated at 37°C for 2 h. The absorbance at 450 nm was measured using a microplate reader (J&H technology Co., Ltd, Jiangsu, China) to determine cell viability.

### Xenograft model and grouping for animal experiments

A C3H xenograft model was established using male C57BL/6 mice (6 weeks, Shanghai Slac, China). Briefly, the mice were injected subcutaneously with 5 × 10^5^ cells/0.1 mL dissolved in 50% Matrigel® (BD Biosciences, New York, USA) at the right flank (n = 5 for each group). The concentration of the exosomes injected into the mice was 2 mg/mL. For the evaluation of the PD-L1 function in the in vivo growth of C3H cells, PD-L1-overexpressing C3H cells were planted into the C57BL/6 mice. The mice injected with normal C3H cells were set as the negative control.

C57/BL/6 mice were planted with normal C3H cells, and exosomes were injected intravenously through the tail vein thrice before and after planting to investigate the effects of exosome-loaded PD-L1 on the in vivo growth of C3H cells. The length (L) and width (W) of each tumor were detected to calculate the tumor size according to the following formula: (*L* × *W*^2^)/2.

### Flow cytometry

A monocyte suspension was obtained from tumor tissues, transferred to EP tubes, and added with an FITC-labeled CD3 antibody. FITC-labeled mouse antihuman IgG was taken as the isotype control. After incubation in the dark for 20 min, the cells were washed with the PBS buffer and loaded onto a flow cytometer (BD, New York, USA).

### Clinical tumor tissue collection and immunohistochemical analysis

The tumor tissues of six clinical patients with OS (3 PD-L1 positive and 3 PD-L1 negative) were collected. PD-L1-negative and PD-L1-positive OS tissues were screened through immunohistochemical analysis. Briefly, the tissues were fixed with 4% paraformaldehyde, dehydrated, and embedded in paraffin. Subsequently, they were cut into 5 μm sections, deparaffinized, and rehydrated. Afterward, they were successively incubated with 5% BSA for 30 min, primary antibody against PD-L1 (R&D, Minnesota, USA), and HRP-conjugated secondary antibody. Lastly, images were taken using a light microscope (Laird, Missouri, USA). After the PD-L1-negative and PD-L1-positive OS tissues were screened, the exosomes were separately extracted from these tissues.

### Statistical analysis

Data were expressed as mean ± SD and analyzed using the GraphPad software. Data between two groups were examined via Student’s t-test, and data among groups were evaluated through one-way ANOVA. Differences were considered significant when p < 0.05.

## Results

The immune evasion mechanism mediated by exosomes carried by PD-L1 in OS was investigated to explore its potential as a therapeutic target for OS treatment. This study was performed to investigate the effects of PD-L1-loaded exosomes on the tumor growth of OS. Exosomes were extracted from cells and tissues through ultracentrifugation. IFN-γ production was determined to evaluate the activity of Jurkat cells. The in vivo growth of OS cells was evaluated with a C3H xenograft model. Tumor volumes were monitored, and the proportion of CD3+ T cells was detected in the tumor tissues.

### PD-L1 was highly expressed in OS cell lines

**Figure 1.**
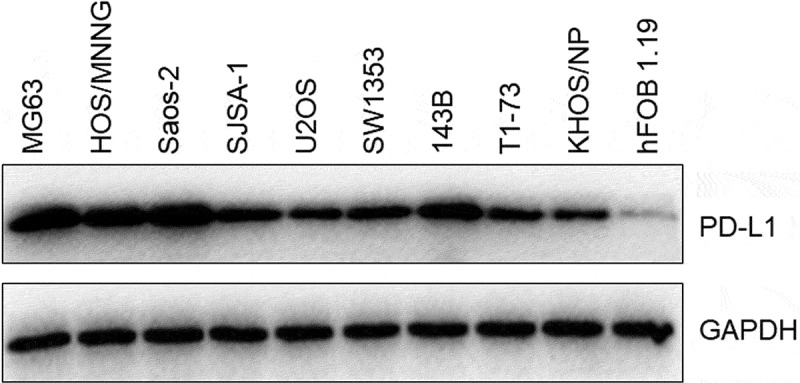
The expression level of PD-L1 in MG63, HOS/MNNG, Saos-2, SJSA-1, U2OS, SW1353, 143B, T1-73, KHOS/NP, and hFOB 1.19 cells was determined by Western blotting assay

PD-L1 expression levels were determined in nine different types of OS cell lines and one type of normal human osteoblasts (hFOB1.19) to confirm the expression level of PD-L1 in OS cell lines and screen the optimized OS cell lines. As shown in Figure 1, PD-L1 was significantly upregulated in OS cell lines compared with that in hFOB1.19 cells. Among the cells, MG63 and Saos-2 cells had the highest PD-L1 expression. Therefore, they were utilized in the subsequent experiments.

### PD-L1-loaded exosomes were extracted from OS cells

**Figure 2.**
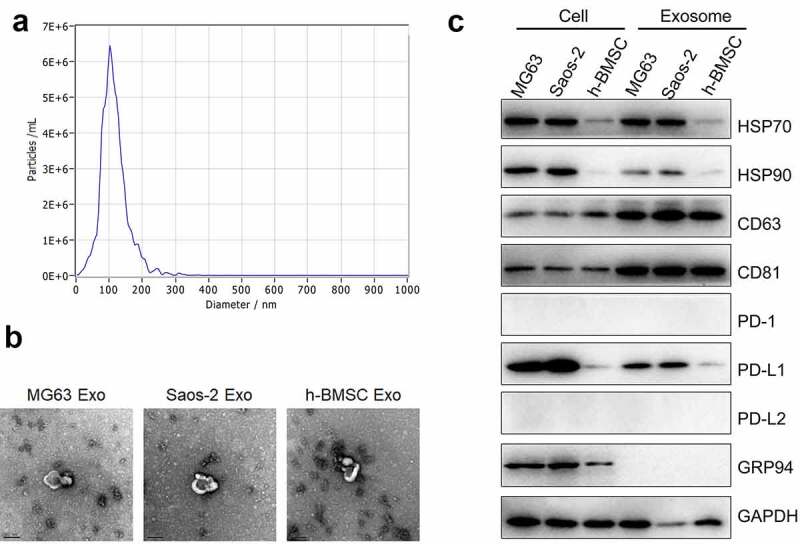
Exosomes were extracted using ultracentrifugation. a. Nanoparticle tracking analysis was used to measure the distribution of particle size (60,000×). b. The ultrastructure of exosomes was visualized by TEM. c. The expression level of HSP70, HSP90, CD63, CD81, PD-1, PD-L1, PD-L2, GRP 94 and GAPDH was detected by Western blotting assay

The exosomes were then extracted from MG63 and Saos-2 cells through ultracentrifugation. The ultrastructure and size distribution are presented in Figures 2a and b. The average diameter of the extracted exosomes was around 100 nm, which was within the diameter range of previously reported exosomes. Exosome biomarkers, such as Hsp70, Hsp90, CD63, and CD81, were subsequently detected. Hsp70 and Hsp90 were observed in the cell lysis and the exosomes (Figure 2c). The expression levels of CD63 and CD81 in the exosomes were higher than those in the cell lysis. PD-1 and PD-L2 were almost not expressed in all the cell lines, and the PD-L1 expression in the exosomes was proportional to that in the cells. Therefore, the exosomes extracted from OS cells were loaded with PD-L1.

### T cell activity was inhibited by PD-L1-loaded exosomes

**Figure 3.**
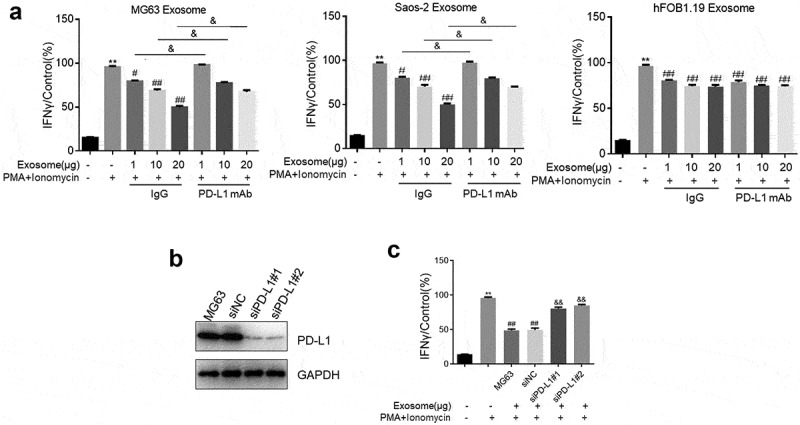
Exosomes extracted from OS cells inactivated Jurkat cells through its loaded PD-L1. a. The production of IFN-γ was determined in MG63 Exosome, Saos-2 Exsome, and hFOB1.19 Exosome by ELISA assay (p < 0.05, p < 0.01). b. Western blotting assay was used to evaluate the expression level of PD-L1 in Jurkat cells treated with PBS, siNC, siPD-L1#1, and siPD-L1#2. c. The secretion of IFN-γ from MG63 cells with or without Exosome and PMA+ionomycn treatment was measured by ELISA assay (p < 0.01)

Jurkat cells were activated when PMA and ionomycin were stimulated. Their activation was represented by the detected IFN-γ secretion. Jurkat cells were incubated with PMA and ionomycin in the absence or presence of exosomes (1, 10, and 20 μg) supplemented with IgG or PD-L1 mAb. In Figure 3a, IFN-γ production was significantly increased by the stimulation of PMA and ionomycin compared with that of the control (Jurkat cells without stimulation and exosome treatments), but it was dramatically decreased by the treatment of MG63 exosomes or Saos-2 exosomes (p < 0.05 or p < 0.01). IFN-γ secretion was greatly increased by the cotreatment of exosomes and PD-L1 mAb compared with that of the Jurkat cells treated with exosomes supplemented with IgG (p < 0.05). Interestingly, the increase in the IFN-γ concentration in the activated Jurkat cells was significantly suppressed by the co-introduction of IgG and hFOB1.19 exosomes. However, no difference was observed in the groups added with PD-L1 mAb compared with that in the IgG groups. Dose-dependent IFN-γ production was not observed in hFOB1.19 exosome-treated Jurkat cells.

Two siRNAs against hPD-L1 were designed and transfected into MG63 cells to obtain PD-L1-knocked down MG63 cells and to further verify whether the activity of Jurkat cells was suppressed by MG63 exosomes through the function of PD-L1. The exosomes were extracted, and the PD-L1 expression level was determined. PD-L1 was significantly downregulated in the exosomes isolated from siPD-L1#1-transfected MG63 cells and siPD-L1#2-transfected MG63 cells compared with that in the siNC group (Figure 3b). Subsequently, IFN-γ production was detected after Jurkat cells were incubated with PMA and ionomycin in the absence or presence of exosomes isolated from different cells (MG63 cells, MG63 cells transfected with siNC, MG63 cells transfected with siPD-L1#1, and MG63 cells transfected with siPD-L1#2). In Figure 3c, compared with the control, IFN-γ secretion was significantly elevated by the stimulation of PMA and ionomycin, which was greatly decreased by the introduction of MG63 exosomes. IFN-γ production was dramatically increased by the exosomes isolated from MG63 cells transfected with siPD-L1#1 or MG63 cells transfected with siPD-L1#2 compared with that in the siNC group (p < 0.01).

### The growth of OS cells was facilitated by PD-L1

**Figure 4.**
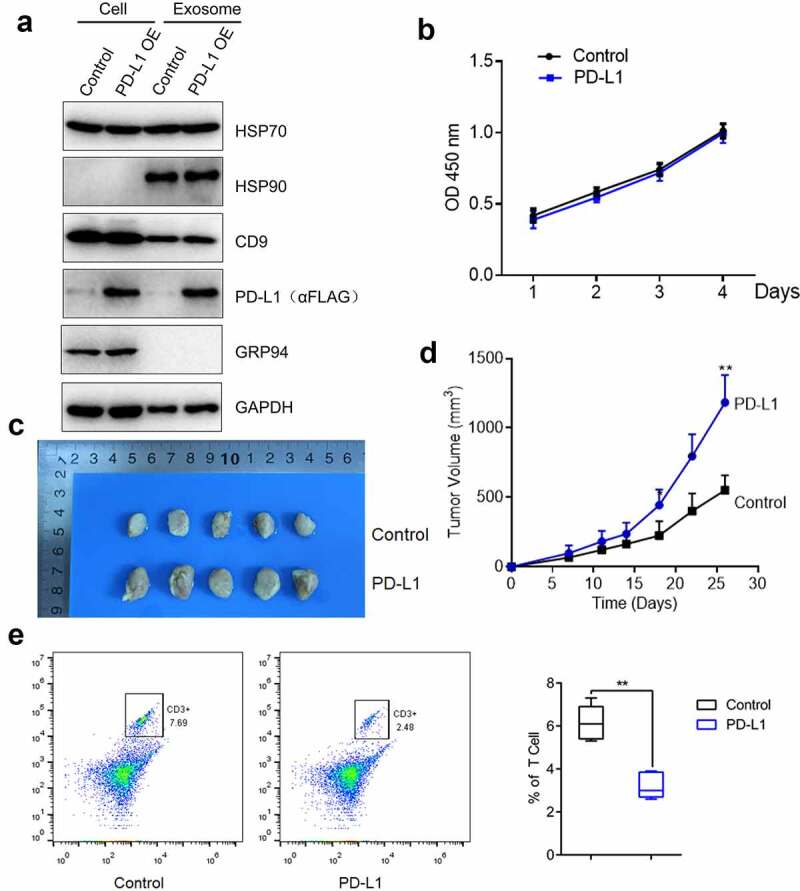
PD-L1 induced the growth of OS cells. a. The expression level of HSP70, HSP90, CD9, PD-L1, GRP 94 and GAPDH was determined by Western blotting. b. Cell viability was measured by CCK-8 assay. c-d. The in vivo growth of OS cells was evaluated by xenograft model (p < 0.01). e. Flow cytometry was used to determine the proportion of CD3 + T cells in the tumor tissues (p < 0.01)

The function of PD-L1 in OS cell proliferation was further explored. In this part, C3H cells were used. mPD-L1-overexpressing C3H cells were established, and exosomes were extracted. In Figure 4a, PD-L1 was highly expressed in PD-L1-transfected C3H cells and the exosomes extracted from these cells. Subsequently, the proliferation of C3H cells was not influenced by PD-L1 overexpression (Figure 4b). Afterward, normal C3H cells and PD-L1-overexpressing C3H cells were planted into the C57/BL/6 mice, and the tumor volume was monitored for nearly 1 month. In Figures 4c and d, the tumor volume of the mice injected with PD-L1-overexpressing C3H cells (p < 0.01) was significantly larger than that of the control. This result indicated that the in vivo growth of OS cells was significantly facilitated by PD-L1 overexpression. Lastly, the monocyte suspension was obtained from the tumor tissues, and the proportion of CD3 + T cells was detected by flow cytometry. In Figure 4e, the proportion of CD3 + T cells in the tumor tissues of the mice injected with PD-L1-overexpressing C3H cells significantly decreased compared with that of the control (p < 0.01). Therefore, the decreased infiltration of CD3 + T cells in tumor tissues was induced by PD-L1 overexpression.

### In vivo growth of OS cells was facilitated by PD-L1-loaded exosomes

**Figure 5.**
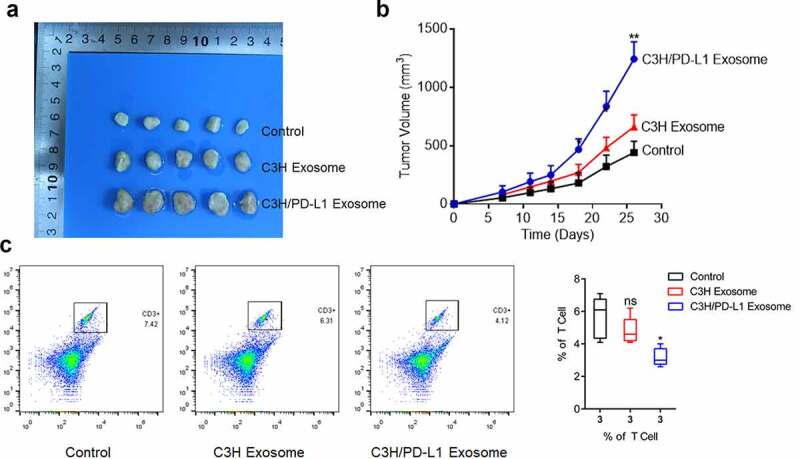
Exosomal PD-L1 induced the growth of OS cells. a-b. The in vivo growth of OS cells was evaluated by xenograft model (p < 0.01). c. Flow cytometry was used to determine the proportion of CD3 + T cells in the tumor tissues (p < 0.05)

The exosomes were extracted from normal C3H cells and PD-L1-overexpressing C3H cells to explore whether the effects of PD-L1 on tumor growth could be mediated by tumor-derived exosomes. The exosomes were administered to the C3H cell-planted C57BL/6 mice. In Figures 5a and b, the tumor volume was slightly elevated in the C3H exosome group compared with that in the control group and significantly increased in the PD-L1-overexpressing C3H exosome group. This result indicated that the tumor growth was significantly facilitated by PD-L1-loaded exosomes (p < 0.01). In addition, the proportion of CD3 + T cells (Figure 5c) was dramatically decreased in the PD-L1-overexpressing C3H exosome group compared with that in the C3H exosome group (p < 0.05). Therefore, the infiltration of CD3 + T cells was significantly suppressed by PD-L1-loaded exosomes.

### The role of PD-L1-loaded exosomes was verified in clinical tissues

**Figure 6.**
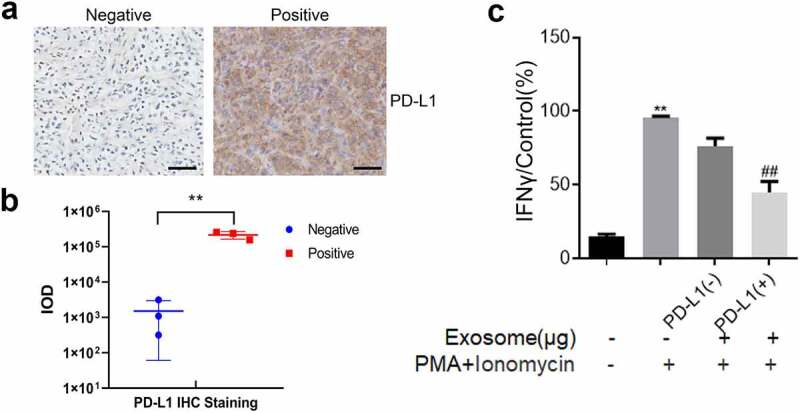
Exosomes extracted from PD-L1 positive OS tissues inactivated Jurkat cells. a. Immunohistochemical assay was used to determine the expression level of PD-L1 in the clinical tumor tissues. The Bar length was 25 μm. b. Relationship between the amount of PD-L1-positive exosomes and the expression of PD-L1 in tumor tissues. c. The production of IFN-γ was determined by ELISA assay (p < 0.01)

Clinical OS tissues were collected to evaluate PD-L1 expression and ensure the consistency of PD-L1-loaded exosome function in murine models and humans. In Figure 6a, the PD-L1-positive and PD-L1-negative OS tissues were separated through an immunohistochemical assay. Subsequently, the exosomes were extracted from these tissues. The results indicated that the relative PD-L1 expression in exosomes was consistent with that in OS tissues (Figure 6b). Lastly, Jurkat cells were incubated with the exosomes extracted from PD-L1-positive and PD-L1-negative OS tissues. The findings showed that the high IFN-γ production (Figure 6c) was slightly decreased by the treatment of exosomes derived from PD-L1-negative OS tissues and significantly decreased by the treatment of exosomes derived from PD-L1-positive OS tissues (p < 0.01). Therefore, the inhibitory effect of PD-L1-loaded exosomes on T cell activity was also observed in human OS tissues.

## Discussion

The immune system plays a critical role in the elimination of tumor cells. However, malignant tumors have several mechanisms of immune escape to prevent from being attacked by the immune system [21,22]. Immune checkpoints are a group of negative regulators of the immune system, and tumors evade the immune system by creating an inhibitory tumor microenvironment through the induction of the upregulation of immune checkpoints [23,24]. Among these checkpoints, PD-1 suppresses the activity of immune cells by binding to its ligand, PD-L1 [25]. PD-L1 is highly expressed in multiple types of malignant tumors, including OS [26,27]. After the binding between PD-1 and PD-L1, immunoreceptor tyrosine-based inhibition motif is phosphorylated to recruit the phosphatases SHP1 and SHP2, which further induce the dephosphorylation of the downstream molecules of T cell receptors (TCRs), such as CD3 and ZAP70, to terminate TCR signaling activation [28,29]. Effector T cell response includes the expression of the proinflammatory cytokine IFN-γ. Huang conducted a meta-analysis and found 14%–80.6% positive distribution of PD-1/PD-L1 in patients with OS; they also observed a positive correlation between tumor metastasis and PD/PD-L1 signaling [30]. HLA-I-negative/PD-L1-positive tumors are larger (T) and have a lower grade of infiltration with CD8 + T cells [31]. Besides, the average number of tumor-infiltrating CD8+ and CD45RO+ T cells is lower in PD-L1-positive tissues than in PD-L1-negative tissues [32]. PD-1-PD-L1 blockade has been shown to reverse tumor-induced T cell exhaustion/dysfunction in patients with colorectal cancer [33]. In Jurkat T cells co-cultured with IFN-γ-stimulated NCC24 and YCCEL1 cells, the number of cells in the G0/G1 phase is significantly increased [34]. This G0/G1 arrest is partially released by administering anti-PD-L1 antibody. The combination of anti-PD-1 antibody and cisplatin significantly inhibited the in vivo tumor growth [35]. T cells induced the PD-L1 expression in primary gastric adenocarcinoma epithelial cells in an IFN-γ-dependent manner, thereby promoting T cell apoptosis [36]. However, this effect is reversed by blocking PD-L1. Consistent with the high level of PD-L1 in OS in a previous report [37], the upregulation of PD-L1 in OS cells in our study was verified in nine different OS cell lines. The effects of PD-L1 were investigated by transfecting C3H cells with mPD-L1 plasmids. Although the in vitro proliferation of C3H cells was not affected by mPD-L1 overexpression, their in vivo growth was significantly facilitated by mPD-L1 overexpression and accompanied by the decreased infiltration of CD3 + T cells. These data verified the promoting effect of PD1/PD-L1 signaling in OS progression [38]. PD-L1 mAb treatment differs in various cell lines likely because of the varied response of different cell lines. Therefore, PD-1/PD-L1 signaling may be different in various cell lines.

Exosomes are a group of vesicles with a diameter of around 100 nm, and they play a critical role in communication among different cell types [13]. The effects of exosomes derived from tumor cells on immune escape have been widely reported. Time- and dose-dependent inhibitory effects on the proliferation ability and production of cytokines are observed in the coculture medium of CD3+ Jurkat cells and exosomes from ovarian carcinoma cells [39]. In a clinical report, exosomal PD-L1 is considered a candidate biomarker of the pulmonary metastasis progression of patients with OS [40]. This study provided evidence that exosomes derived from OS cells contributed to cancer progression by loading with PD-L1. The effects of PD-L1-loaded exosomes on OS progression were further verified in the C3H xenograft model of C57BL/6 mice. In addition, the exosomes extracted from clinical PD-L1-positive OS tissues showed a promising inhibitory property against activated T cells. Therefore, the role of PD-L1 in mice was consistent with that in humans.

## Limitation

In our study, the inhibitory effect of exosomes derived from OS cells on T cell activation was significantly abolished by the antibody against PD-L1 or by a specific siRNA targeting PD-L1. This result indicated the promising immune suppression property of PD-L1-loaded OS cell exosomes. Interestingly, exosomes derived from normal osteoblasts also elicited inhibitory effects on T cell activation, but this process was not affected by the treatment of PD-L1 antibody.

In our future work, we will investigate mechanisms other than PD-L1, such as miRNAs or lncRNAs, which mediate immune suppression functions in exosomes.

## Conclusion

Exosomes extracted from clinical PD-L1-positive OS tissues showed a promising inhibitory property against activated T cells. Therefore, PD-L1-loaded exosomes extracted from OS cells aggravated OS progression by suppressing T cell activities.

## Highlights

- PD-L1 was significantly upregulated in OS cell lines.
- PD-L1-overloaded exosomes significantly facilitated OS cell growth.
- Exosomes extracted from clinical PD-L1-positive OS tissues inhibited T cell activation.
- PD-L1-loaded exosomes aggravated OS progression.
